# Metabolism of Cannabidiol in Respiratory-Associated Cells and HepG2-Derived Cells and Molecular Docking of Cannabidiol and Its Metabolites with CYP Enzymes and Cannabinoid Receptors

**DOI:** 10.3390/ijms26178384

**Published:** 2025-08-28

**Authors:** Krittawan Tongkanarak, Pijush Kumar Paul, Muhammad A. Khumaini Mudhar Bintang, Roongnapa Suedee, Somchai Sawatdee, Teerapol Srichana

**Affiliations:** 1 Drug Delivery System Excellence Center, Department of Pharmaceutical Technology, Faculty of Pharmaceutical Sciences, Prince of Songkla University, Hat Yai, Songkhla 90112, Thailand; krittawan.t@psu.ac.th (K.T.); alikhumaini31@gmail.com (M.A.K.M.B.); 2 Department of Nutrition and Health Sciences, University of Nebraska-Lincoln, Lincoln, NE 68583, USA; pijushpaulgb@gmail.com; 3 Molecular Recognition Materials Research Unit, Drug Delivery System Excellence Center, Department of Pharmaceutical Chemistry, Faculty of Pharmaceutical Sciences, Prince of Songkla University, Hat Yai, Songkhla 90112, Thailand; roongnapa.s@psu.ac.th; 4 Drug and Cosmetics Excellence Center and School of Pharmacy, Walailak University, Thasala, Nakhon Si Thammarat 80161, Thailand; somchai.sa@wu.ac.th

**Keywords:** cannabidiol, cannabinoid receptor, metabolism, CYP450, drug delivery

## Abstract

Cannabidiol (CBD) has been reported in medical applications for various indications. The enzymatic metabolism of CBD is not fully understood in the different routes of administration. This research aimed to identify the CBD metabolites after incubation of CBD with derived hepatocyte cells (HepG2), bronchial epithelial cells (NCI-H358), alveolar cells (A549), and alveolar macrophage cells (NR8383). A liquid chromatography–mass spectrometry technique was developed to quantify the CBD and its metabolites. Molecular docking was employed to evaluate the binding affinity of CBD with different cytochrome P-450 (CYP-450) enzymes and further predict the implication of drug–drug interactions. CBD and major metabolites of CBD were also docked with cannabinoid receptors. The results revealed that only HepG2 cells metabolized CBD to 7-hydroxy-CBD (7-OH-CBD) and 7-carboxy-CBD (7-COOH-CBD), whereas other respiratory cell lines and alveolar macrophages were found to have mainly CBD in the incubated samples without any metabolites. The CYP2C19 and CYP3A4 enzymes were responsible for CBD conversion to hydroxylated CBD metabolites. The 7-OH-CBD and 7-COOH-CBD metabolites were found to bind to cannabinoid receptors with different affinities. The relative abundance of CBD and major metabolites may indicate the potential route of CBD administration.

## 1. Introduction

**Figure 1:**
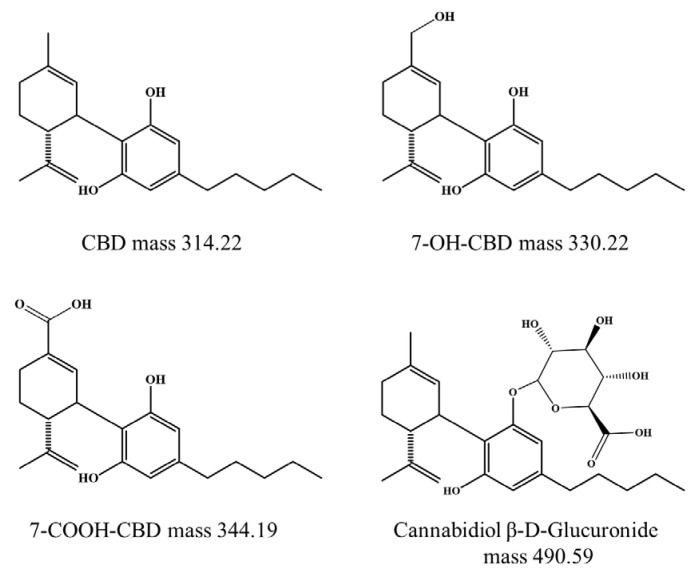
Molecular mass of CBD and other reported metabolites in Daltons [8].

Cannabidiol (CBD) and tetrahydrocannabinol (THC) are the major phytocannabinoids in cannabis, which has a variety of potentially pharmacological activities that include anti-inflammatory, anticonvulsant, antiemetic, antioxidant, and anxiolytic effects [1]. The human body has two cannabinoid receptors: cannabinoid 1 (CB1) and cannabinoid 2 (CB2) [2]. CB1 receptors are predominantly present in the central nervous system whereas CB2 receptors are mainly in the peripheral system. THC can bind with CB1 receptors that are expressed in the brain and are associated with motor control, emotional responses and behaviors. However, THC has limitations due to its psychotic effects. On the other hand, CBD binds with CB2 receptors and lacks psychoactivity from the low affinity of binding with CB1 receptor [1,3]. CBD is metabolized in vitro as well as in vivo in experimental animals and humans [4,5]. The metabolic reactions of CBD are primarily in the liver where CBD undergoes cytochrome P450 (CYP-450) enzyme contribution [1]. The main metabolic transformations of CBD involve allylic hydroxylation, specifically at the 6- and 7-positions. Among the CYP enzymes expressed in the liver, CBD plays a substrate role and is metabolized mainly by CYP3A4 and to a lesser extent by CYP2C19 [6]. The results are supported by a pharmacokinetic interaction study and the pharmacological relevance of CBD-ketoconazole and CBD-omeprazole that are inhibitors of CYP3A4 and CYP2C19, respectively [1]. These enzymes play a crucial role in converting CBD into a range of metabolites, some of which may have biological activity and therapeutic potential and may have different pharmacological properties than CBD itself [7,8]. CBD undergoes hepatic metabolism to form 7-hydroxy-CBD (7-OH-CBD) as a primary active intermediate that is then further converted to the more polar 7-carboxy-CBD (7-COOH-CBD), which is an inactive metabolite at carbon 7 (Figure 1). These metabolites are formed by hydroxylation and carboxylation by CYP-450 [4]. Glucuronidation occurs to conjugate the CBD molecule by UDP-glucuronosyltransferase enzymes for CBD excretion as CBD-glucuronide [9]. Hence, a gain of metabolites that have a higher affinity to CB1 and CB2 may therefore have greater therapeutic potential [8]. The route of administration can influence the metabolism and excretion of CBD. Furthermore, the pharmacokinetics of CBD showed highly variable individuality due to the limited bioavailability and drug–drug interactions when taking CBD concomitantly with CYP450 substrates. The therapeutic potential of oral CBD is limited because the bioavailability typically ranges from 4% to 20% due to the low solubility in water of CBD [10]. This is attributed to irregular absorption and extensive first-pass hepatic metabolism [11]. Following oral administration, 7-COOH-CBD becomes the primary circulating metabolite with a significantly observed high area under the concentration–time curve that is 40 times greater than CBD in the blood circulation [10]. Higher oral CBD doses that range from 5 to 25 mg/kg or from 350 to 1750 mg/day are often required to achieve therapeutic blood levels while drug concomitant administration and toxicity related to the high dose need to be considered [8,12].

The respiratory system is an important route of administration for inhaled drugs to reach higher systemic concentrations, which includes cannabinoids. When inhaled, CBD is rapidly absorbed into the bloodstream and can interact with the endocannabinoid system specifically with the CB2 receptor, which regulates various physiological processes including inflammation and immune functions [13]. Earlier research explored the delivery of CBD by heating and vaporizing the CBD dose before inhalation [14]. CBD showed benefits in the treatment of inflammatory lung diseases by decreasing pro-inflammatory cytokines and chemokines in the bronchoalveolar space [15]. The inhalation of CBD has shown rapid detection in the plasma and brain which is required for immediate treatment. In a rat study, inhalation of CBD via vaporizer (20 mg per group of four rats) led to immediate detection of CBD in the bloodstream with measurable concentrations that appeared in the brain within 15 min [16,17]. However, complex delivery systems face limitations due to the equipment involved and challenges associated with patient self-administration, particularly in urgent and time-sensitive situations. Therefore, achieving effective pulmonary delivery of CBD formulations requires rapid onset and high bioavailability of CBD. However, the metabolism of inhalable CBD in the respiratory system has not been thoroughly investigated [18].

Pre-systemic metabolism of CBD in the lung can be different from liver metabolism because of differences in cellular functions and enzymatic activity of the airways and liver cells. However, extrahepatic metabolism that occurs in the lungs has received comparatively little attention. The lung epithelium expresses several CYP-450 enzymes, such as CYP1A2 and CYP3A5, which may contribute to local drug metabolism [19]. Understanding the metabolism of CBD in respiratory-associated cell lines can have important implications for its therapeutic potential. Identification of metabolites and enzymes involved in metabolism could help to elucidate the pharmacokinetics of CBD and its potential therapeutic effects [16]. This information can be used to optimize dosing strategies and develop new CBD formulations for inhalation.

This study aimed to tentatively characterize the metabolic profile of CBD following incubation with liver-derived cells, respiratory epithelial cell lines, and alveolar macrophages. A validated liquid chromatography–mass spectrometry (LC-MS/MS) method was developed and applied to quantify key CBD metabolites. Molecular docking was conducted to further explore the metabolic pathways and assess the binding affinity of CBD with various CYP enzymes. In addition, the major identified metabolites were docked with cannabinoid receptors to evaluate their potential interactions and biological relevance.

## 2. Results

### 2.1. Cell Viability Assay

**Figure 2:**
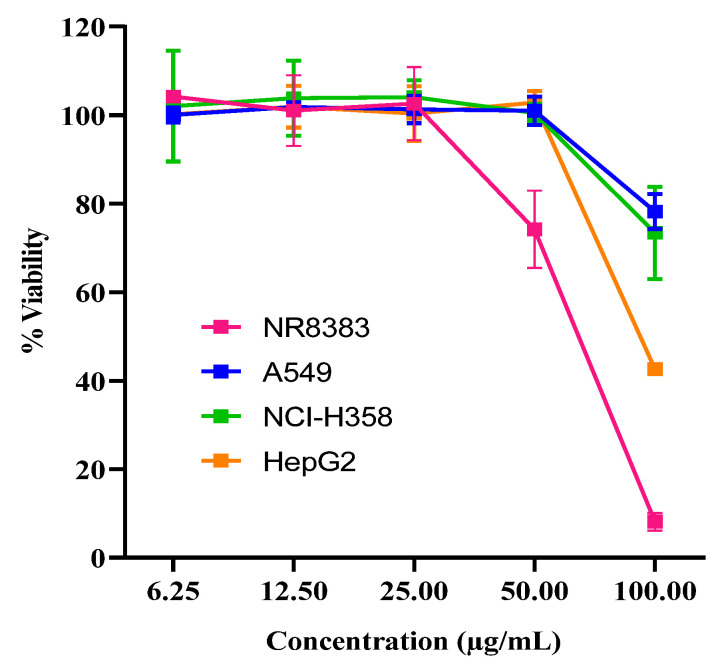
Viabilities of NR8383, A549, and NCI-H358 respiratory cell lines and derived hepatocytes (HepG2) during exposure to the standard CBD at concentrations of 6.25–100 µg/mL (mean ± SD, *n* = 3).

Figure 2 shows the cell viability of different cell lines exposed to CBD at concentrations of 6.25–100 µg/mL. CBD at concentrations of 100 µg/mL was toxic to all cell lines. At CBD concentrations of 25 µg/mL or lower, all cell lines exhibited viabilities above 95%, which indicated concentrations lower than 25 µg/mL in the assay were appropriate to conduct further CBD metabolism studies.

### 2.2. Analysis of CBD by LC-MS/IT-TOF

**Figure 3:**
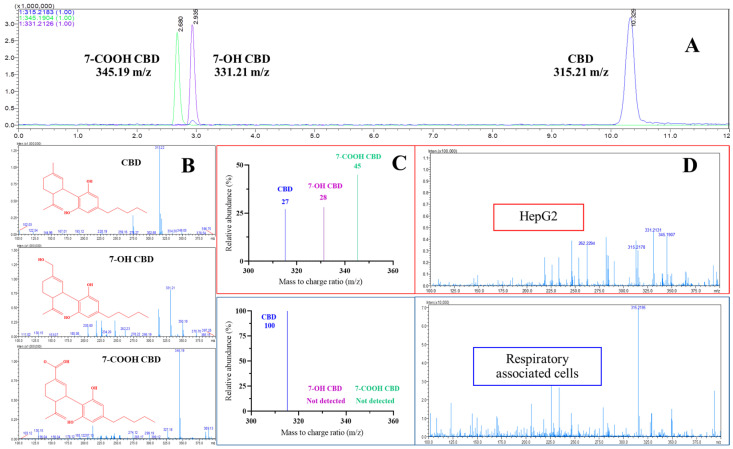
Chromatogram, percent relative abundance and mass spectra in LC-MS/MS positive ESI mode (centroid) of CBD at *m*/*z* = 315.21 and its metabolites at *m*/*z* = 331.21 (7-OH-CBD) and *m*/*z* = 345.19 (7-COOH-CBD) (**A**–**C**) derived from the incubation of CBD in Hep-G2 and respiratory associated cell lines for 24 h (**D**).

The developed method was run to identify precursor standard masses for structure confirmation and quantification. The calibration curve was linear from 10 µg/mL to 50 µg/mL with a high correlation coefficient (r^2^ = 0.9998). The limit of detection and limit of quantification were found to be 0.79 µg/mL and 2.41 µg/mL, respectively. From the chromatographic analyses of the standard CBD, 7-OH-CBD, and 7-COOH-CBD, the backgrounds were subtracted from the solvent to obtain accurate results. The elution times of the standard CBD, 7-OH-CBD, and 7-COOH-CBD were found to be 10.3, 2.9, and 2.7 min, respectively (Figure 3A). Mass analysis of the CBD peak revealed a molecular ion (positive; M^+^) of 315.21 *m*/*z*, which was confirmed to be the standard peak with protonation of CBD while 7-OH-CBD and 7-COOH-CBD were shown at 331.21 *m*/*z* and 345.19 *m*/*z*, respectively (Figure 3B).

### 2.3. CBD Metabolism in HepG2 Cells and Lung-Associated Cells

When the MS spectra results were compared between the standard CBD and the sample extracted from the HepG2 cells and lung-associated cells, differences in *m*/*z* were found. The standard CBD showed the most abundant *m*/*z* in positive electrospray ionization (ESI^+^ mode) at 315.21 *m*/*z*, which is CBD, while extraction of the CBD from the HepG2 cells showed abundant *m*/*z* values of 331.21 *m*/*z* as 7-OH-CBD and 345.19 *m*/*z* as 7-COOH-CBD while CBD at 315.21 *m*/*z* was observed to be lower compared to its standard at 24 h after incubation. The percent relative abundances of CBD, 7-OH-CBD, and 7-COOH-CBD from HepG2 cells were determined from their amount ratios and found to be 27%, 28%, and 45%, respectively (Figure 3C). The observation of 7-OH-CBD and 7-COOH-CBD after incubation was related to the hepatocytic cytochrome mechanism, a CYP-mediated activation of xenobiotics to metabolites which reported 7-OH-CBD was converted by CYP3A4 and CYP2C19 from CBD while the metabolism of 7-COOH-CBD was further converted from 7-OH by CYP3A4 [20]. The observed mass indicated that the CBD metabolites were modified by the HepG2 cells in accordance with hepatocytic metabolism [21]. CBD metabolism in lung-associated cells was studied. The differences in gene expression among different lung cell lines has been reported in metabolically active cells [1]. The three respiratory cells which reported metabolic capability with detectable CYP-450 were selected to represent an aspect of how individual cells within the lung complexity respond to the cellular metabolism due to its unique characteristics [22,23]. In this study specific cell types were selected for accurate assessment. The bronchial cells are represented by the NCI-H358 cell line, which is used to gain a better understanding of the impact of substances on lung health. The NCI-H358 cells are epithelial-like cells that were isolated from the bronchiole of a male patient with bronchioalveolar carcinoma. Expression of the monooxygenase CYP-448 and other xenobiotic-metabolizing enzyme activities was found in NCI-H358 [24].

After deposition of large particles on the bronchial cells, smaller particles are transported further to the alveolar cells. A549 cells represent alveolar cells in the alveoli zone of the human lung. The A549 cell line is a human lung adenocarcinoma cell line that is widely used in research, particularly in lung cancer studies, drug discovery, and toxicology research. It was derived from a carcinoma of the lung of a 58-year-old white male. These cells are a model for studying lung cancer biology, developing drug therapies, and assessing the effects of toxic substances. The A549 cell line is a valuable tool for researchers due to its ability to maintain some of the characteristics of type II alveolar epithelial cells. Several CYP-450 enzymes are expressed in A549 cells where they participate in metabolic inactivation and activation of numerous exogenous and endogenous compounds [25]. The NR8383 cell line represents alveolar macrophages (immune cells) which are the first to detoxify any agent reaching the lung. The results showed that only CBD as a parent mass was detected from all lung-associated cell lines, which indicated that CBD was unlikely to be metabolized (Figure 3D).

### 2.4. Binding Interaction by Molecular Docking

**Figure 4:**
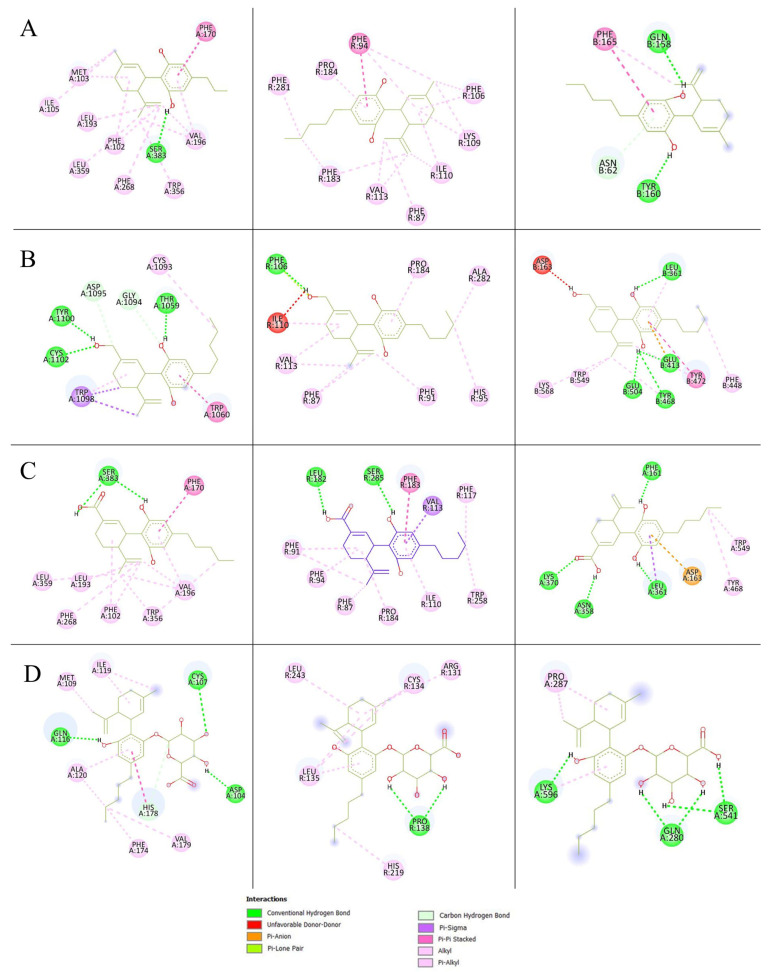
Principal interactions of (**A**) CBD, (**B**) 7-OH-CBD, (**C**) 7-COOH-CBD, and (**D**) CBD-G of the highest energy against the CB1 receptor (**left**), CB2 receptor (**middle**), and β-glucuronidase (**right**).

Molecular docking simulations evaluated the binding affinities of CBD and its metabolites 7-OH-CBD, 7-COOH-CBD, and CBD-G with CB1, CB2, and β-glucuronidase. All re-docked structures had root mean standard deviation (RMSD) values below 2 Å, which indicated reliable docking accuracy. These values are provided in Table 1. The binding interactions are shown in Figure 4 and the docking scores are shown in Table 2. CBD had the highest binding affinity to CB2 (−9.6 kcal/mol) followed by CB1 (−9.38 kcal/mol) and β-glucuronidase (−8.54 kcal/mol). Notably, 7-OH-CBD demonstrated the highest affinity for CB1 (−9.69 kcal/mol) that was likely attributed to additional hydrogen bonding interactions. In contrast, its affinity for CB2 (−9.11 kcal/mol) was slightly lower than CBD. 7-COOH-CBD followed a comparable pattern that exhibited a modest reduction in CB1 binding affinity (−9.01 kcal/mol) while maintaining a slightly stronger affinity for CB2 (−9.16 kcal/mol). The glucuronidase metabolite, CBD-G, displayed markedly weaker binding across all targets (−6.07 to −6.48 kcal/mol), which suggested that glucuronidation diminishes receptor interactions and may serve as a metabolic deactivation pathway.

CBD and its metabolites primarily interact with CB1 and CB2 through hydrophobic contacts, with key residues such as TRP356 (tryptophan) and PHE94 (phenylalanine) contributing to strong binding. 7-OH-CBD exhibited additional hydrogen bonds in CB1 that reinforce its stability. 7-COOH-CBD followed a similar interaction trend but showed slightly reduced binding. CBD-G displayed minimal receptor interactions, which suggested significantly weakened pharmacological activity. For β-glucuronidase, CBD and its oxidative metabolites formed stable hydrogen bonds, whereas CBD-G exhibited weaker interactions, implying inefficient hydrolysis. These findings suggest that hydroxylation and carboxylation retain receptor activity, whereas glucuronidation reduces affinity that likely facilitates systemic clearance [26].

The docking results of CBD on the major metabolized CYP450 enzyme showed several hydrophobic interactions of CBD with the amino acids with the CYP enzyme. The docking scores of the CYP enzymes revealed the estimated affinities between CBD and the bound enzyme with significant differences between CBD binding to CYP1A2 and CYP3A5 and CBD binding to CYP2D6 and CYP2C19 (*t*-test, *p* < 0.01). However, the results showed no statistical differences between the binding of CYP2D6 and CYP1A2 to CBD and the binding of CYP2C19 and CYP3A5 to CBD (Table 3). The atom coordinates of the docking results are provided in Supplemental data as PDB files. Based on the results from the LC-MS/MS methods, a summary of the CBD metabolites in derived hepatocytes and respiratory associated cell lines are presented in Table 4. A total of two metabolites were detected and identified in the HepG2 cells while no metabolite was detected in the respiratory cells. It is important to note that the observed *m*/*z* value of 315.21 represents the molecular ion (M^+^) of CBD.

## 3. Discussion

The use of cannabis has risen steadily in the past decade, which has led to expanded legalization of cannabis for both medicinal and recreational use in many countries [9]. However, a lack of understanding exists regarding the metabolic profile of CBD and how CBD metabolites potentially associate with the therapeutic utility of CBD [27]. In this study, we aimed to develop LC-MS/MS-based methods to screen for major metabolites with closely similar polarities for further metabolic analysis of CBD in different cell lines. From the HepG2 cell line analysis, the fragment with a *m*/*z* value of 331.22 was 7-OH-CBD, which is the primary metabolite of CBD by CYP2C19 metabolism. The 7-OH-CBD metabolite can be further metabolized by CYP3A4 to form an inactive metabolite, which is 7-COOH-CBD with a mass to charge ratio of 345.19. Hepatic enzymes demonstrated a clear metabolic conversion of CBD into 7-OH-CBD and 7-COOH-CBD, which is consistent with a previous report [28]. This suggests that patients who take CBD concomitantly with other drugs may need to be concerned with CYP-mediated drug interactions.

Our experimental data showed that CBD metabolites (OH-CBD and COOH-CBD) were produced by HepG2 liver cells but not detected in other lung-derived cell lines. The results can be hypothesized that the respiratory airway cell lines lack the enzymatic capacity to convert CBD. The results were related to the amount of CYP3A4 and CYP2C19 which were reported in the lower limits of expression in A549 cells [25]. Furthermore, the regulation of CYP3A4 in the human respiratory tract is limited due to lower tissue enriched transcription factors [29]. The capacity of enzymes to mediate CBD metabolism that led to the conversion of CBD to its metabolites was specific to the hepatic cell line. To the best of our knowledge, this study provides evidence suggesting that respiratory cell lines are not involved in the generation of major metabolites from CBD. Nevertheless, additional research is required to comprehensively comprehend the metabolism of CBD within the respiratory system and its potential implications for both effectiveness and safety. CBD activates both CB1 and CB2 receptors with higher affinity to CB2, which exhibit anti-inflammatory, immunomodulatory, and analgesic properties [30]. CB2 receptors are primarily found in the peripheral organs, particularly in immune system cells. By modulating the immune system, the CB2 receptor regulates the release of inflammatory cytokines. Molecular docking simulation was utilized to understand the potential biological roles of these metabolites and determine whether CBD and metabolites could activate the CB1, CB2 and β-glucuronidase receptor. Blind docking was employed. CBD and its metabolites were allowed to explore the entire receptor surface. While the lowest-energy poses were located in the orthosteric site (particularly for CB2), several conformations were also observed in potential allosteric regions. Notably, these results are consistent with the allosteric sites of CB1 and CB2 described in previous studies [31,32], supporting the possibility that CBD may act as an allosteric modulator [33].To evaluate the relative binding affinities the most stable binding poses were reported for consistency.

The results showed CB2 displayed higher affinity for CBD than the 7-COOH-CBD and 7-OH-CBD metabolites. The hydroxyl part of CBD metabolites was shown to form hydrogen bonding and the lower affinity of the metabolites came from the hydrophobic nature of the binding pocket of the CB2 receptor. Among the metabolites, the findings revealed that 7-COOH-CBD exhibited a higher capability to dock into the CB2 binding pocket with minimal steric hindrance compared to 7-OH-CBD. Moreover, in this study CBD was docked against the major CYP isoforms. Docking helps predict whether the metabolites are still able to bind and modulate these proteins. For example, docking of inositol hexaphosphate (IP6) to CYP1A2 showed that IP6 binds poorly in the heme pocket [1]. The docking results indicate that binding energies are largely driven by hydrophobic contacts. CBD is metabolized mainly by CYP2C19 and CYP3A4 in hydroxylation reactions that occur at aliphatic pentyl- and propenyl-substituted CBD. Given that the primary metabolism of CBD predominantly occurs through its hydrophobic region, it is preferable for CBD to bind to more hydrophobic sites to initiate hydroxylation reactions. This preference is observed in the results obtained with CYP2C9, CYP2C19, and CYP3A4. Across both CB1 and CB2 complexes, the highest-affinity poses showed extensive nonpolar interactions in the receptor pockets. This finding is in agreement with past studies, which showed that hydrophobic interactions play a major role in the strong binding affinity observed in both CB receptors [2]. While numerous docking studies have explored the interaction of parent CBD with various biological targets, very few have examined CBD metabolites, especially 7-OH-CBD, 7-COOH-CBD, or CBD-G, despite their biological relevance. A recent study by Rao et al. [34] performed docking of CBD-derived metabolites to the CB1 receptor and observed generally poor binding for most metabolite forms, which suggested reduced activity. However, their work did not include docking to CB2 receptors, CYP enzymes, or β-glucuronidase, nor did it focus specifically on 7-OH- or 7-COOH-CBD, which are the key phase I oxidative metabolites. The docking results help interpret whether these metabolites retain potential pharmacological activity (e.g., 7-OH-CBD binding to CB2), are likely inactivated (e.g., CBD-glucuronide), or might interact with metabolic enzymes. This structure–function correlation provides mechanistic insight into CBD’s metabolic fate and complements our experimental findings on metabolite distribution in pulmonary and hepatic cells.

## 4. Materials and Methods

### 4.1. Materials

A CBD sample was provided gratis by Quantum Biotech Ltd. (Pathum Thani, Thailand) with 99% purity. A CBD standard was obtained from the Department of Medical Sciences, Ministry of Public Health (Bangkok, Thailand). Standards of 7-OH-CBD and 7-COOH-CBD were obtained from Sigma-Aldrich (St. Louis, MO, USA). Analytical grade absolute ethanol and acetonitrile were acquired from RCI Labscan (Bangkok, Thailand). Human lung adenocarcinoma cell line (A549), alveolar macrophage cell line (NR8383), human bronchioalveolar carcinoma cell line (NCI-H358), human hepatocellular carcinoma cell line (HepG2), and the solutions related to maintaining the cell cultures were obtained from the American Type Culture Collection (ATCC, Rockville, MD, USA). 3-(4,5-dimethylthiazol-2-yl)-2,5-diphenyltetrazolium bromide (MTT) was from Sigma-Aldrich (St. Louis, MO, USA).

### 4.2. Chemical Analysis of CBD

The CBD analysis was conducted using a high-performance liquid chromatography (HPLC) system (CBM-20A; Shimadzu Corporation, Tokyo, Japan) as previously described [35]. The separations were carried out on a C18 reverse-phase column (150 mm × 2.1 mm, 3 µm, Hypersil BDS; Thermo Fisher Scientific, MA, USA) at a temperature of 15 °C. The mobile phase was a mixture of 70% acetonitrile and 30% ultrapure water. The flow rate was set to 0.5 mL/min, and an injection volume of 20 µL was used. Spectrophotometric monitoring was performed at a wavelength of 207 nm. The obtained data were processed using LabSolutions™ Version 3.81.418 (Shimadzu Corporation, Tokyo, Japan).

### 4.3. Liquid Chromatography–Tandem Ion Trap Time-of-Flight Mass Spectrometry (LC-MS/IT-TOF) of CBD and CBD Metabolites

The LC-MS/MS method was developed as a high throughput and highly sensitive quantitative analysis of CBD and its metabolites. The LC-MS/MS system consisted of a LC system (controller CBM-20A, pumps LC-30AD, and auto-sampler SIL-30AC; Shimadzu, Tokyo, Japan) with an ion-trap/time-of-flight (IT-TOF) mass spectrometer (Shimadzu, Tokyo, Japan). The HALO C18 column (2.1 mm id × 150 mm, 2.7 µm particle size; Thermo Fisher Scientific, Scoresby, Australia) was used with 0.1% formic acid in acetonitrile and Milli-Q water in a 70:30 ratio by volume as the mobile phase at a flow rate of 0.2 mL/min. The ion spray voltage, collision energy, curved desolvation line, and nebulizing gas were set to 1.70 kV, 50%, 200 °C, and 1.50 L/min, respectively. The mass spectrometer was equipped with the electrospray ionization interface operated in the positive ion mode in the range of *m*/*z* of 100–400 for 15 min with an ultra-small volume injection of 1 µL due to the mass sensitivity. Data analysis and acquisition were performed using ACD/Labs software (Version S05S41).

### 4.4. Cell Line Conditions

#### 4.4.1. Alveolar Macrophage Cells

The NR8383 cell line was cultured in F-12 Kaighn’s medium (Gibco^®^, Waltham, MA, USA) supplemented with 15% fetal bovine serum (FBS, Gibco^®^, Waltham, MA, USA) and 100 U/mL penicillin/streptomycin (Gibco^®^, Waltham, MA, USA). The cells were incubated at 37 °C in a 5% CO_2_ incubator, and the culture medium was changed every 2 days. A gentle rocking motion was applied to harvest the cells, and fresh culture medium was added to create a new single-cell suspension for subsequent incubation.

#### 4.4.2. Human Alveolar Cells

The A549 cell line was cultured in Kaighn’s Modification of Ham’s F-12 Medium (F-12K, Gibco^®^, Waltham, MA, USA) supplemented with 10% fetal bovine serum, and 100 U/mL penicillin/streptomycin. The culture was maintained under 5% CO_2_ at 37 °C. The culture medium was refreshed every other day. Once the cells reached confluence, they were harvested using 0.25% trypsin-EDTA. Subsequently, fresh culture medium was added to create a new single-cell suspension for further incubation.

#### 4.4.3. Human Bronchioalveolar Carcinoma Cells

The NCI-H358 cell line was cultured in RPMI-1640 Medium (Gibco^®^, Waltham, MA, USA) supplemented with 10% fetal bovine serum and 100 U/mL penicillin/streptomycin. The cells were incubated at 37 °C in a 5% CO_2_ incubator, and the medium was changed every 2 days. A gentle rocking motion was applied to harvest the cells, and fresh culture medium was added to create a new single-cell suspension for subsequent incubation.

#### 4.4.4. Human Hepatocellular Carcinoma Cell Line

The HepG2 cell line was cultured in Eagle Minimum Essential Medium (MEM, Gibco^®^, Waltham, MA, USA) containing 10% fetal bovine serum and antibiotics (100 U/mL penicillin/streptomycin) under 5% CO_2_ at 37 °C. The culture medium was refreshed every other day. Once the cells reached confluence, they were harvested using 0.25% trypsin-EDTA. Subsequently, fresh culture medium was added to create a new single-cell suspension for a certain period of time (log phase culture: 4 days).

#### 4.4.5. Cell Proliferation and Viability Assay

One hundred microliters of the NR8383, A549, NCI-H358, and HepG2 cell lines at concentrations of 1 × 10^5^ cell/mL were seeded in a 96-wells plate in complete media. After 24 h, the CBD standard at concentration ranges of 6.25–100 µg/mL in fresh medium was added into the culture plates. Cells without the CBD served as negative controls. After incubation for 24 h, a MTT solution (5 mg/mL) assay was performed to evaluate cell activity. Briefly, the cells were treated with 80 µL of fresh media along with 20 µL of MTT solution and incubated at 37 °C under 5% CO_2_ for 4 h. Thereafter, media containing MTT were removed and 100 µL of dimethyl sulfoxide was added. The absorbance was determined by a microplate reader (Biohit 830, Biohit^®^, Helsinki, Finland) at a wavelength of 570 nm. The percentage of cell proliferation was calculated and compared to the negative control.

### 4.5. Drug Metabolism

After the HepG2, A549, NCI-H358, and NR8383 cell lines were grown for 4 days, the cultures were treated with the CBD standard at a nontoxic concentration of 12.5 µg/mL (Figure S1) in complete media and incubated at 37 °C in 5% CO_2_ for 24 h. After incubation, the cultured cells were treated with 0.2 mL of 0.1% sodium dodecyl sulfate (SDS, Sigma, USA). The cell culture flasks were incubated at 37 °C for 3 min to lyse the cells. After incubation, 0.8 mL of complete medium was added to the culture flasks and filtered through a 0.22 mm nylon membrane filter. The culture were kept in a freezer at −20 °C until assay.

### 4.6. Liquid–Liquid Extraction of CBD and Metabolites from the Cell Culture

The liquid–liquid phase was used to extract the sample from the cell culture before analysis of the drug and metabolites. The lysed cell samples in culture media from Section 4.5. at 1 mL were thawed at room temperature. One milliliter of sample was added into 3 mL of extraction solvent (ethyl acetate) and then vortexed for 3 min. The culture medium was aspirated. The organic layer containing the drug and metabolites was transferred to a new tube. The extraction process was repeated 2 times to increase the CBD extraction efficiency. The supernatant was evaporated to dryness with a stream of oxygen-free nitrogen at room temperature. The residue was reconstituted with 1 mL of LC-MS/MS mobile phase, and a 1 µL sample was injected into the liquid chromatography system with LC-MS/IT-TOF analysis.

### 4.7. Molecular Docking Prediction

Protein structures were obtained from the RCSB Protein Data Bank by selecting entries that represented biologically active conformations with co-crystallized ligands to ensure the relevance of binding site geometry for molecular interaction studies. For the CB1 and CB2, structures bound to known ligands were used to capture their active or ligand-bound conformations. The β-glucuronidase structure was selected based on its use in previous drug metabolism and prodrug activation studies. The CYP-450 isoforms (CYP1A2, CYP2C9, CYP2C19, CYP2D6, CYP3A4, and CYP3A5) were chosen based on their established roles in CBD metabolism and availability of structures complexed with substrates or inhibitors, reflecting catalytically competent states. Statistical analysis was performed using GraphPad Prism 8 software. A *p*-value less than 0.01 was considered significant. Docking was performed using AutoDock 4.2 software obtained from the Scripps Research Institute (http://autodock.scripps.edu/ (accessed on 31 January 2025)) [36]. The CBD file as ligand was obtained from PubChem (ID: 644019) and docked to six different CYP450 enzymes that acted as macromolecules obtained from rscb.org, which were 1A2 (ID: 2HI4), 2C9 (ID: 1R9O), 2C19 (ID: 4GQS), 2D6 (ID: 5TFT), 3A4 (ID: 1TQN), and 3A5 (ID: 7SV2). All ligands, water molecules, and non-essential heteroatoms were removed from the macromolecules. The PDB file obtained was then converted to pdbqt (a molecule with partial charges and atom type) using the AutoDock Tools script for ligand and macromolecule. The ligand and receptor files were loaded on the AutoDock Tools software to prepare a grid parameter file (.gpf). This involved setting up a grid box that encloses the macromolecule in its default position for blind docking. The docking parameter file (.dpf) was prepared by setting the number of samples to 200 (population size) and setting the number of runs to 50 on the genetic algorithm search parameter and using Lamarckian GA as the output. The docking was then processed in AutoDock Tools using the pdbqt file of the receptor and ligand grid parameter files and the docking parameter file to produce the docking log file (.dlg). The resultant files were then analysed and the lowest docking score conformations were analysed and visualized in Discovery Studio Visualizer Software Version 25.1.0.24284 (BIOVIA, Dassault Systèmes^®^, San Diego, CA, USA). To understand the interaction between CBD and its metabolites with CB1, CB2, and β-glucuronidase, molecular docking studies were performed using AutoDock 4.2 as mentioned previously to compare binding affinities and key interactions. In this case, the ligands were obtained from PubChem, which were CBD (ID: 644019), 7-COOH-CBD (ID: 146592489), 7-OH-CBD (ID: 11301963) and CBD-G (drawn and optimized in Avogadro), docked into the CB1 (id:5TGZ), CB2 (ID: 6KPF) and β-glucuronidase (id:3LPG). Native co-crystallized ligands were re-docked into their original protein structures to validate the docking protocol. The RMSD values were computed using AutoDockTools. Alignment between the docked and reference poses confirmed the reliability of the docking setup.

## 5. Conclusions

The metabolic conversions of CBD to 7-OH-CBD and 7-COOH-CBD were observed exclusively in derived hepatocyte cells, while other respiratory cell lines and alveolar macrophages predominantly showed the presence of CBD in the incubated samples without significant formation of major metabolites that were seen in the HepG2 cell line. The oral formulation may encounter CBD metabolism to inactive metabolites by the liver pathway. Inhalation is suggested to be an alternative route. Notably, CBD, as well as its 7-OH-CBD and 7-COOH-CBD metabolites, demonstrated varying affinities when binding to CB2 receptors. Further investigations will help to clarify the precise mechanism of CBD metabolism in the respiratory tract and the binding interaction of the CBD metabolites with CB2 receptors which may result in the bioactivity of the metabolites.

## Figures and Tables

**Table 1:** RMSD values from re-docking validation of native ligands into their respective protein binding sites.

Target Protein	PDB ID	RMSD (Å)
CB1	5TGZ	1.936
CB2	6KPF	0.952
β-Glucuronidase	3LPG	1.794
CYP1A2	2HI4	0.571
CYP2C9	1R9O	1.421
CYP2C19	4GQS	1.462
CYP2D6	5TFT	0.882
CYP3A4	1TQN	0.986
CYP3A5	7SV2	0.650

**Table 2:** Docking scores of CBD and its metabolites against CB1, CB2, and β-glucuronidase.

Ligand	Macromolecule		
	CB1 (kcal/mol)	CB2 (kcal/mol)	β-Glucuronidase (kcal/mol)
CBD	−9.38	−9.60	−8.54
7-OH-CBD	−9.69	−9.11	−8.53
7-COOH-CBD	−9.01	−9.16	−7.89
CBD-G	−6.07	−6.07	−6.48

**Table 3:** Binding energy values of CBD docked on different CYP450 enzymes.

Ligand–Enzyme Interaction	Binding Energy (kcal·mol^−1^)
CBD-CYP1A2	−9.38 ± 0.05
CBD-CYP2D6	−9.31 ± 0.10
CBD-CYP3A5	−8.85 ± 0.07
CBD-CYP2C19	−8.64 ± 0.09

**Table 4:** Summary of CBD metabolites in phase I metabolism from derived hepatocytes and respiratory associated cell lines.

Cell Lines	Observed *m*/*z*	Predicted Formula	Identification
HepG2	315.21	C_21_H_30_O_2_	CBD
	331.21	C_21_H_30_O_3_	7-OH-CBD (hydroxylation)
	345.19	C_21_H_28_O_4_	7-COOH-CBD (oxidation)
NCI-H358	315.21	C_21_H_30_O_2_	CBD
A549	315.21	C_21_H_30_O_2_	CBD
NR8383	315.21	C_21_H_30_O_2_	CBD

## Data Availability

The raw data supporting the conclusions of this article will be made available by the authors on request.

## Supplementary Materials

The following supporting information can be downloaded at: https://www.mdpi.com/article/10.3390/ijms26178384/s1.

## Abbreviations

The following abbreviations are used in this manuscript:

CBD	Cannabidiol
CBD-G	Cannabidiol-glucuronide
CB1	Cannabinoid 1 receptor
CB2	Cannabinoid 2 receptor
CYP	Cytochrome
LC-MS/IT-TOF	Liquid Chromatography–Tandem Ion Trap Time-of-Flight Mass Spectrometry
PHE	Phenylalanine
RMSD	Root mean standard deviation
TRP	Tryptophan
THC	Tetrahydrocannabinol
