# Supplementary material for: Metabolism of Cannabidiol in Respiratory-Associated Cells and HepG2-Derived Cells and Molecular Docking of Cannabidiol and Its Metabolites with CYP Enzymes and Cannabinoid Receptors
Source: Int J Mol Sci. 2025 Aug 28;26(17):8384. doi: 10.3390/ijms26178384 (PMC12428432; doi:10.3390/ijms26178384)
Supplement: Supplementary file 1 [file ijms-26-08384-s001.zip › ijms-3797971-supplementary.pdf]

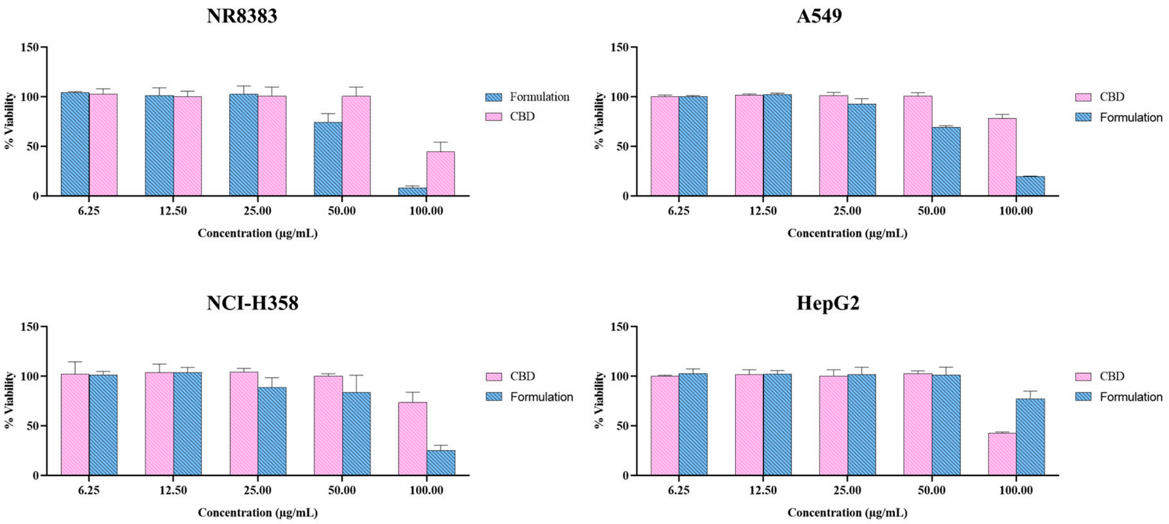

Figure S1. Viabilities of NR8383, A549, and NCI-H358 respiratory cell lines and derived hepatocytes (HepG2) during exposure to the standard CBD and formulation at concentrations of 6.25–100 μg/mL (mean ± SD, n = 3).
